# A novel training simulator for portable ultrasound identification of incorrect newborn endotracheal tube placement – observational diagnostic accuracy study protocol

**DOI:** 10.1186/s12887-019-1717-y

**Published:** 2019-11-13

**Authors:** Hasan S. Merali, Mark O. Tessaro, Khushboo Q. Ali, Shaun K. Morris, Sajid B. Soofi, Shabina Ariff

**Affiliations:** 1 Division of Pediatric Emergency Medicine, McMaster Children’s Hospital, McMaster University, 1280 Main Street West, HSC-2R104, Hamilton, ON L8S 4K1 Canada; 2 Division of Pediatric Emergency Medicine, Emergency Point-of-Care Ultrasound Program, Hospital for Sick Children, 555 University Avenue, Toronto, ON M5G 1X8 Canada; 3 Department of Paediatrics & Child Health, Aga Khan University, Stadium Road, Karachi, 74800 Pakistan; 4 Division of Infectious Diseases and Centre for Global Child Health, Hospital for Sick Children, Department of Pediatrics Faculty of Medicine, 555 University Avenue, Toronto, ON M5G1X8 Canada

**Keywords:** Newborn resuscitation, Point of care ultrasound (POCUS), Simulation, Pakistan, Endotracheal tube (ETT), Intubation

## Abstract

**Background:**

Endotracheal tube (ETT) placement is a critical procedure for newborns that are unable to breathe. Inadvertent esophageal intubation can lead to oxygen deprivation and consequent permanent neurological impairment. Current standard-of-care methods to confirm ETT placement in neonates (auscultation, colorimetric capnography, and chest x-ray) are time consuming or unreliable, especially in the stressful resuscitation environment. Point-of-care ultrasound (POCUS) of the neck has recently emerged as a powerful tool for detecting esophageal ETTs. It is accurate and fast, and is also easy to learn and perform, especially on children.

**Methods:**

This will be an observational diagnostic accuracy study consisting of two phases and conducted at the Aga Khan University Hospital in Karachi, Pakistan. In phase 1, neonatal health care providers that currently perform standard-of-care methods for ETT localization, regardless of experience in portable ultrasound, will undergo a two-hour training session. During this session, providers will learn to detect tracheal vs. esophageal ETTs using POCUS. The session will consist of a didactic component, hands-on training with a novel intubation ultrasound simulator, and practice with stable, ventilated newborns. At the end of the session, the providers will undergo an objective structured assessment of technical skills, as well as an evaluation of their ability to differentiate between tracheal and esophageal endotracheal tubes. In phase 2, newborns requiring intubation will be assessed for ETT location via POCUS, at the same time as standard-of-care methods. The initial 2 months of phase 2 will include a quality assurance component to ensure the POCUS accuracy of trained providers. The primary outcome of the study is to determine the accuracy of neck POCUS for ETT location when performed by neonatal providers with focused POCUS training, and the secondary outcome is to determine whether neck POCUS is faster than standard-of-care methods.

**Discussion:**

This study represents the first large investigation of the benefits of POCUS for ETT confirmation in the sickest newborns undergoing intubations for respiratory support.

**Trial registration:**

ClinicalTrials.gov Identifier: NCT03533218. Registered May 2018.

## Background

Two-thirds of global neonatal deaths occur in just 10 countries and the majority occur in Asia [1]. Pakistan has the highest neonatal mortality of Asian nations with 46.6 deaths per 1000 live births [2].

Early neonatal deaths account for 75% of all neonatal deaths, and preventing these deaths depends on attention to the causes of death that are unique to the first week of life, particularly birth asphyxia [3], which accounts for 23% of global neonatal deaths [1]. If not fatal, birth asphyxia can cause severe neurologic impairment including motor and cognitive disabilities [4].

During the first seconds after birth, radical and rapid cardiopulmonary adaptation to the extra-uterine environment must occur for neonates to survive [5, 6]. The most important physiologic change is establishment of independent respiration [7]. It is during this period that approximately 10% neonates require some level of resuscitation [8], and the more premature the newborn, the higher the likelihood that intubation is required [9]. Tracheal intubation is performed frequently in delivery rooms and Neonatal Intensive Care Units (NICU). Neonatal intubation is a critical and time-sensitive procedure, as failure deprives the highest risk newborns of oxygen [10]. Prompt recognition of inadvertent esophageal intubation is vital to prevent the mortality or permanent neurologic consequences of neonatal hypoxia [11, 12]. Unfortunately, neonatal intubation is challenging due to the unique airway anatomy of newborns, and inadvertent esophageal placement of the ETT is common. In a recent multi-center study examining intubations both in the NICU and delivery room, the authors found that esophageal intubation occurred between 1 and 12% of the time [13]. It occurs with even greater frequency with junior trainees often with delayed recognition and correction [11].

Current methods to detect esophageal intubation are imperfect. Chest auscultation is notoriously unreliable [14], especially during noisy and chaotic neonatal resuscitations, where one study found it requires an average of 77 s [15]. Capnography improves upon clinical exam alone, but requires vigorous tissue oxygenation and cardiopulmonary function, [15] which is not present for most peri-arrest neonates. Capnography is usually performed with a qualitative colorimetric device, which exhibits a sensitivity of only 65% for correct airway placement, thus predisposing many correctly intubated neonates to unnecessary re-intubation attempts during which they experience no oxygen delivery and potential airway trauma [16, 17]. These devices also generate false-positive results if they contact secretions or epinephrine, substances commonly suctioned or delivered through ETTs [18]. Capnographic accuracy is improved with quantitative continuous wave-form monitors, but these are expensive, take up to 30 s to generate a result, and still produce false-negative results in peri-arrest patients.

POCUS of the anterior neck is increasingly used by emergency physicians and anesthesiologists to detect esophageal intubation. It is more accurate and faster than physical examination and capnography, with adult meta-analyses reporting sensitivities of 93–98%, specificities of 97–98%, and an average performance time of 9 s [19, 20]. The technique is easy to learn, with trainees serving as the ultrasound operators in most studies [20]. This technique may be most easily applied in children, with reported sensitivities and specificities of 100%, even in children undergoing chest compressions [21].

POCUS presents significant advantages over physical examination and capnography during resuscitation. Its direct cross-sectional imaging of the neck is not affected by cardiopulmonary function, chest compressions, or environmental noise [22]. Due to its quick result, it also prevents the gastric distention and vomiting that can occur from ventilation through an esophageal ETT [23].

Despite its prevalence in adult and pediatric resuscitations, intubation POCUS has not yet translated to neonatology. Only two studies with small sample sizes exist on the detection of esophageal intubation in neonates using portable ultrasound. A recent Cochrane review on methods to determine ETT position in neonates identified portable ultrasound as a promising modality in need of further study [24]. Neonatology services increasingly utilize POCUS for other assessment and diagnostic applications, making uptake of intubation POCUS feasible in neonatal resuscitations.

This study will determine whether neonatal providers that undergo a two-hour training session with a novel intubation POCUS simulator can then accurately detect tracheal vs. esophageal ETTs using POCUS, and generate a more rapid result than standard-of-care methods. This novel intubation POCUS simulator is inexpensive and can be made from simple materials found commonly throughout the world. This study will provide training to the neonatal providers currently performing standard-of-care methods for ETT localization, regardless of POCUS experience, including attending physicians (staff specialists), junior and senior medical trainees, and senior nurses. This study represents the first step in translating the established benefits of intubation POCUS to the sickest newborns undergoing intubation for respiratory support.

## Methods/design

### Study overview and population

This will be an observational diagnostic accuracy study with a training phase (phase 1) and an assessment phase (phase 2). In phase 1, we will train neonatal providers to detect esophageal versus tracheal ETTs using the intubation POCUS simulator, and then assess their accuracy using evaluation tools. In phase 2, newborns requiring intubation in the NICU or labour room/operating room (LR/OR) at The Aga Khan University Hospital, Karachi (AKUH), will have ETT location assessed by POCUS at the same time as current hospital standard-of-care methods (auscultation, colorimetric capnography, and chest x-ray. The total duration of the study will be 18 months.

The Aga Khan University Hospital in Karachi, Pakistan, is an academic tertiary care hospital with a level III NICU. Each year over 5000 deliveries take place at AKUH and approximately 12% of those newborns are admitted to the NICU, of which approximately 70% will require intubation. In addition, approximately 50% of the admitted newborns are transferred from regional hospitals.

### Recruitment and eligibility criteria

Participants for phase 1 of the study will include healthcare providers who currently perform standard-of-care ETT location assessment in the delivery room or NICU at AKUH. This includes neonatologists, neonatology fellows, pediatric residents and senior nurses. These individuals will be identified by the NICU clinical director. Participation is voluntary and may be withdrawn at any time. A detailed informed consent document will be reviewed with each participant prior to the training session and subsequent testing phase.

For phase 2, any newborn needing intubation in the delivery room or NICU will be eligible for the study. Newborns will be excluded if they have abnormal anatomy of the oropharynx or airway, or if the family declines to consent. Importantly, newborns who require emergency intubation at birth will be receive POCUS during resuscitation, as it will not be possible to obtain prior consent. The family will be approached at a later time when they would be able to offer a deferred informed consent, and will decide to have their newborn’s data either included or withdrawn from the study. Neonates requiring non-emergency intubation will be enrolled only if the family provides prior informed consent.

### Ultrasound simulator

**Fig. 1:**
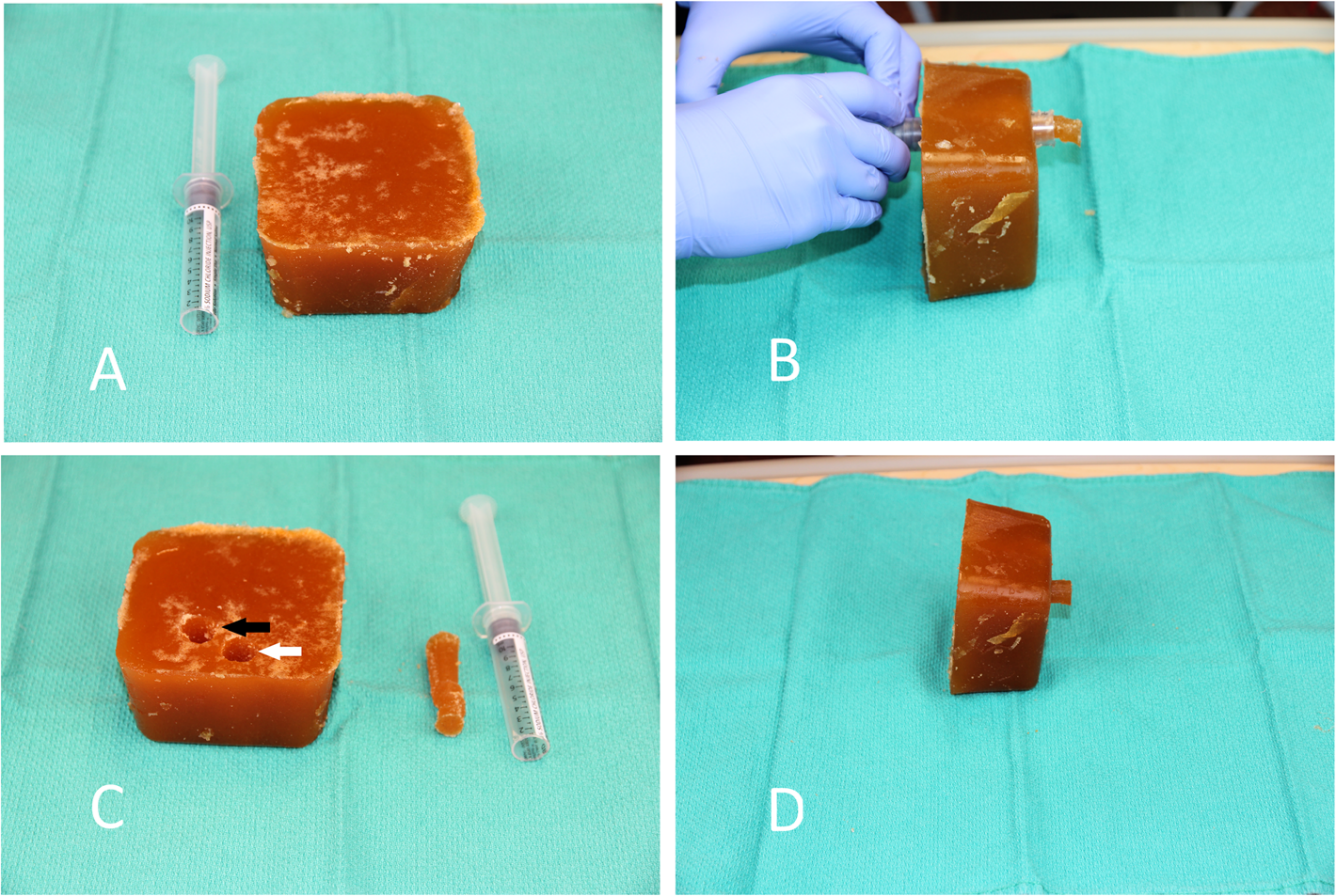
Low-cost ultrasound simulator. **a** The beef gelatine and psyllium fiber block, with cut-off 10 mL syringe barrel. **b** With cut-off 10 mL syringe barrel being used to create staggered hollow cylinders within the block. **c** Block with staggered hollow cylinders, with retained plug from cylinder creation. More superior cylinder is simulated trachea (white arrow), and more inferior cylinder is simulated esophagus (black arrow). **d** Plug partial insertion into more inferior cylinder (model esophagus)

In this study, we will utilize a novel, low-cost airway ultrasound simulator which is made on a stove by mixing beef gelatin powder, orange colored psyllium fiber, and water [25]. It costs approximately $2 USD for the materials required to create the simulator. It then requires overnight refrigeration and a simple procedure using a 5 ml syringe to create a simulated trachea and esophagus (see Fig. 1).

### Ultrasound equipment

In this study, we will utilize the Philips Lumify USB ultrasound system chosen for its compact design, image quality and portability. Specifically, the L12–4 linear array transducer will be utilized. The depth of the probe will be set at 2.5 cm. The probe will be connected to an Android tablet, version 4.5 this system allows for videos between 1 s and 10 min to be captured.

### Intervention – phase 1

**Fig. 2:**
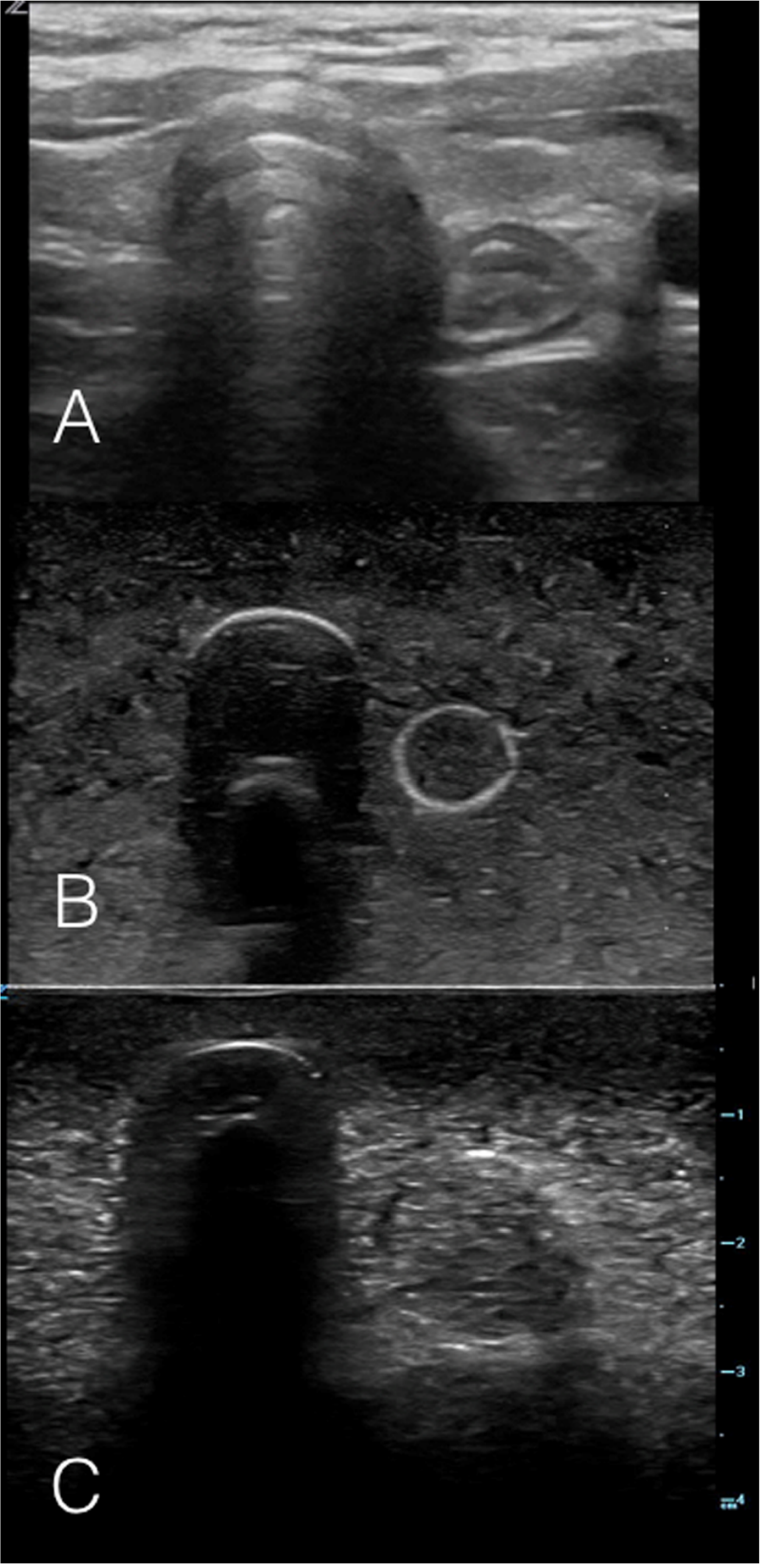
Ultrasound images on the model: Tracheal Intubation. **a** Static ultrasound image of a patient with the ETT in the trachea, with linear ultrasound probe held in transverse orientation over the anterior neck at the level of the sternal notch. **b** & **c** Static ultrasound images of beef gelatine model with plug inserted into simulated esophagus, simulating the ultrasound appearance of a tracheal intubation

**Fig. 3:**
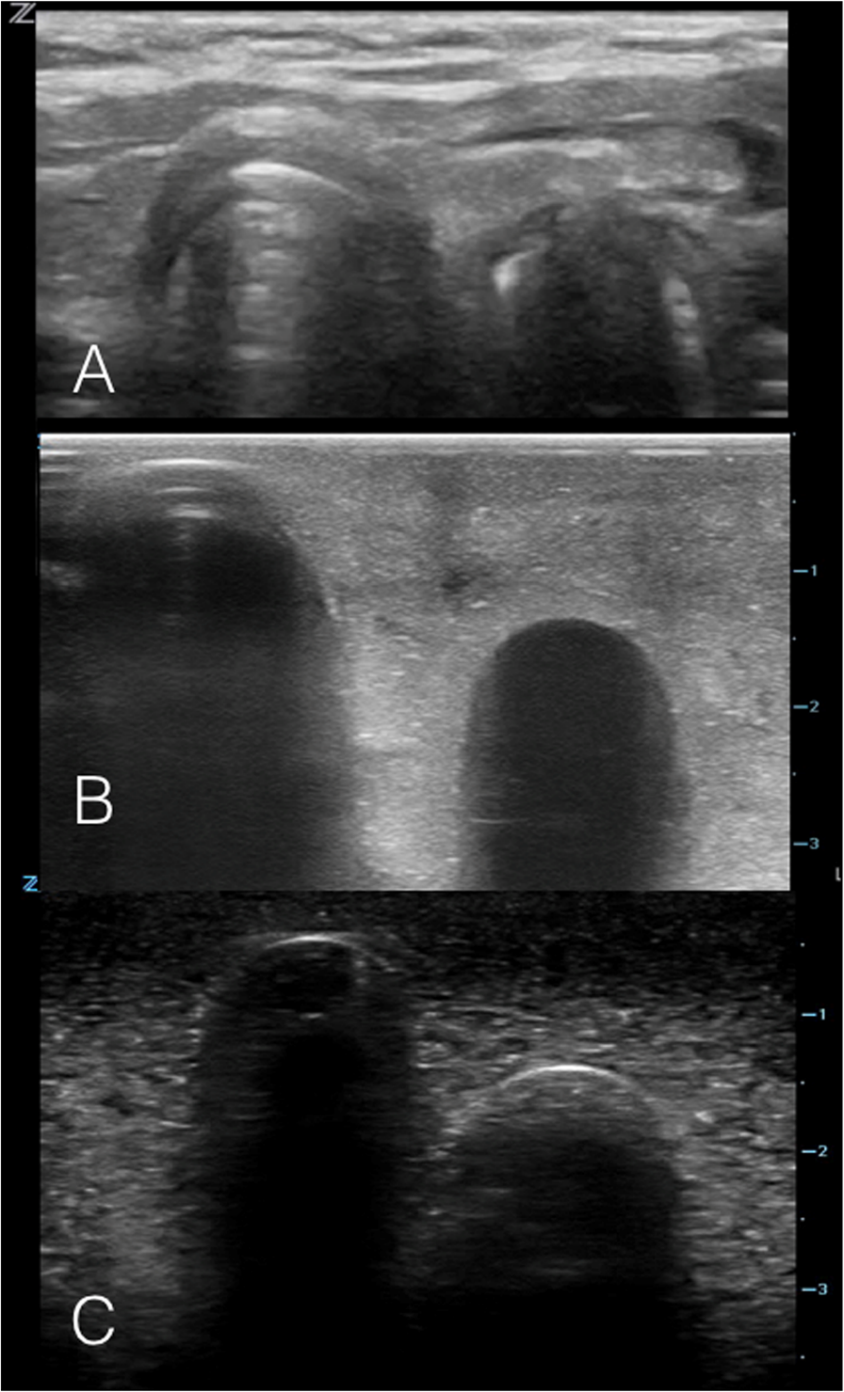
Ultrasound images on the model: Esophageal Intubation. **a** Static ultrasound image of an esophageal intubation in a patient, with linear ultrasound probe held in transverse orientation over the anterior neck at the level of the sternal notch. **b** & **c** Static ultrasound images of beef gelatine model with plug removed from simulated esophagus, simulating the ultrasound appearance of an esophageal intubation

**Fig. 4:**
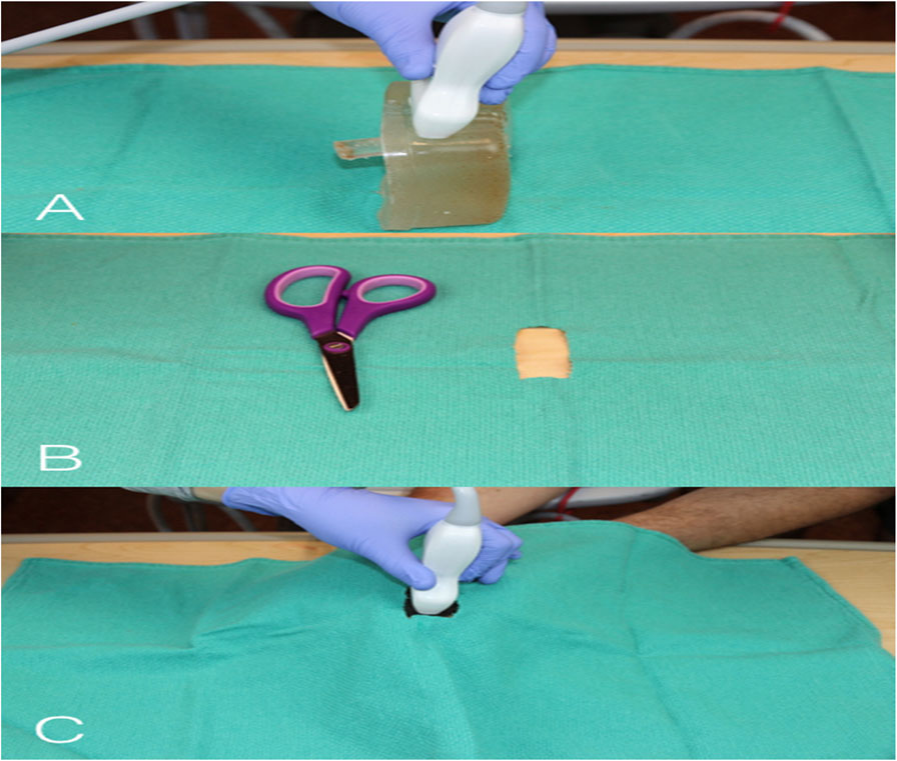
Placement of the probe on the Simulator. **a** The first image shows placement of linear probe over the side of the model to obtain images in transverse plane. **b** Demonstrates a towel with a square hole of 1 X 3 cm and **c** Shows the towel is placed over the beef gelatin model to hide the location of the plug from the ultrasound operator

**Fig. 5:**
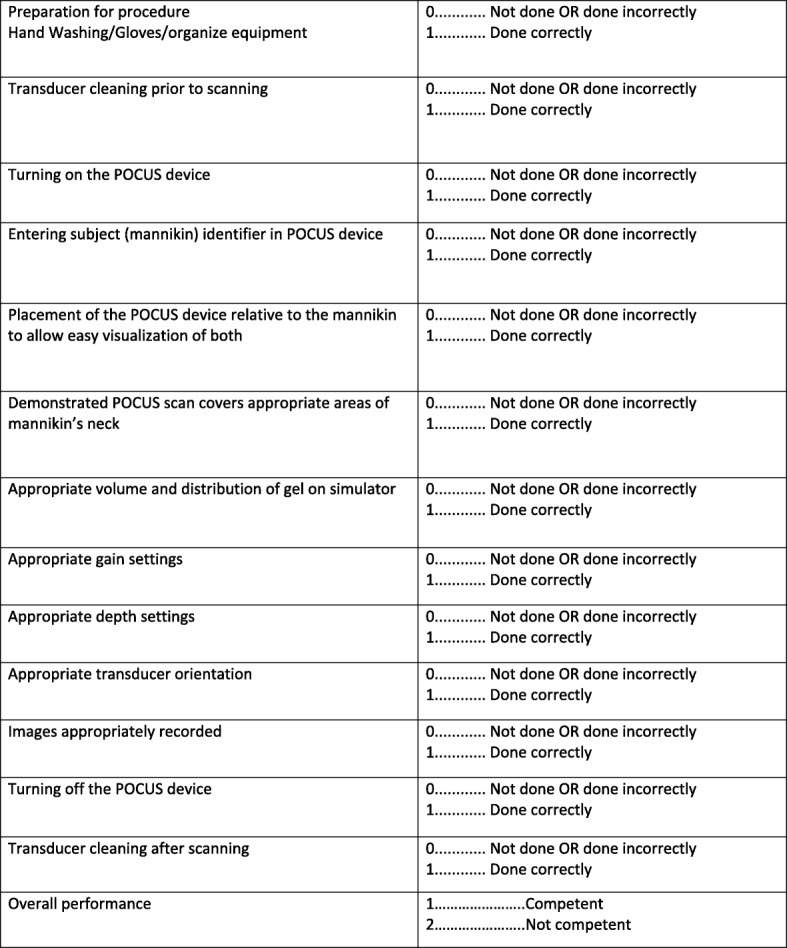
Objective Structured Assessment of Technical Skills

**Fig. 6:**
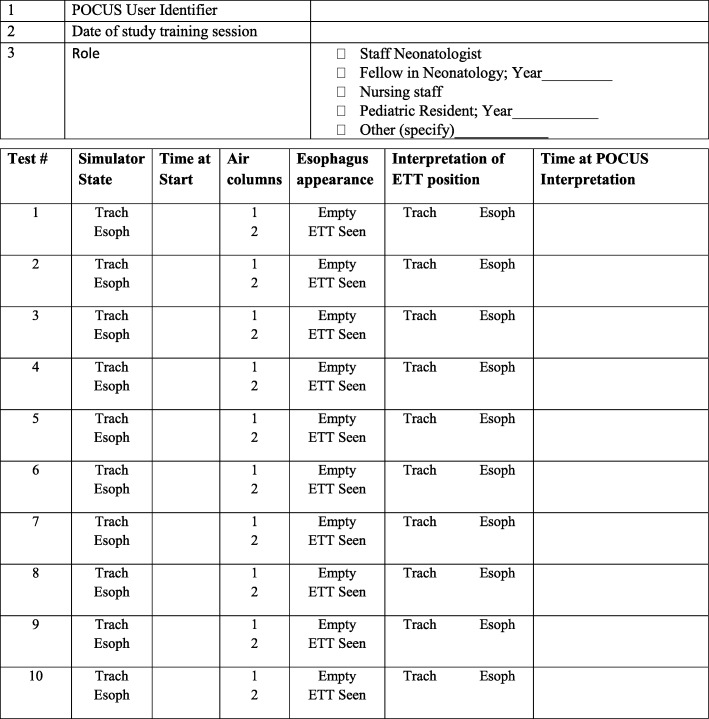
Learner Evaluation Form

The first phase is training of the healthcare providers to sonographically identify correct tracheal and incorrect esophageal placement of an ETT. This two-hour session will be conducted by a study co-investigator with paediatric airway POCUS expertise. It will begin with a didactic presentation, followed by demonstration using a newborn doll (to demonstrate gel use and probe positioning) and the airway ultrasound simulator (to demonstrate POCUS image acquisition and interpretation (see Figs. 2 and 3). After a period of practicing gel use and probe positioning with the newborn doll, the healthcare providers will then demonstrate these same skills on stable intubated and non-intubated patients in the NICU. The instructor will assess these skills and then provide feedback to the healthcare providers. The healthcare providers will then practice ETT location detection using the airway ultrasound simulators (see Fig. 4). Finally, the healthcare providers will undergo an objective structured assessment of technical skills (see Fig. 5), as well as an evaluation of their ability to differentiate between tracheal and esophageal ETTs (see Fig. 6). Only those participants who pass both these examinations will move on to phase 2. Providers who do not obtain passing scores will be allowed to repeat the training course one additional time prior to re-examination.

### Assessment – phase 2

The second phase of this project is an observational diagnostic accuracy study. Newborns at AKUH receiving intubation will be assessed by POCUS at the same time as standard-of-care methods for ETT location (auscultation, colorimetric capnography, and chest x-ray), and the accuracy of the healthcare providers using POCUS will be determined. The time required to assess ETT location will also be compared between POCUS and standard-of-care methods.

There will be 2 POCUS machines available, one in the NICU and the other in the LR/OR. At the time of delivery, a clinical team consisting of a neonatology fellow, a pediatric resident, and a staff nurse are available to receive the newborn. If a newborn requires intubation, this will be performed by the fellow or resident, who will assess tracheal versus esophageal ETT location via standard-of-care methods. The clinical team will be accompanied by an independent research POCUS team, consisting of a POCUS user and a research assistant. The POCUS user will be a healthcare provider who has successfully completed phase 1 of the study.

The research POCUS team will be completely independent of the clinical team and will not provide them with results of the POCUS assessment. After completing the POCUS examination, the POCUS user will announce “complete” (to notify the research assistant for timing purposes) and will leave the room to record their findings. This will ensure that the clinical team is not affected by the result of our research test (which has unknown accuracy in this population). The POCUS user will have 30 s to complete the POCUS examination, after which the research assistant will notify them that their time is up. The research assistant will be present during the entire intubation and will record the time it takes for ETT confirmation via ultrasound and standard-of-care methods. If the patient needs more than one intubation attempt, then this information will be recorded by the research assistant, but the POCUS exam will only be performed with the first attempt.

### Data management and quality control

**Fig. 7:**
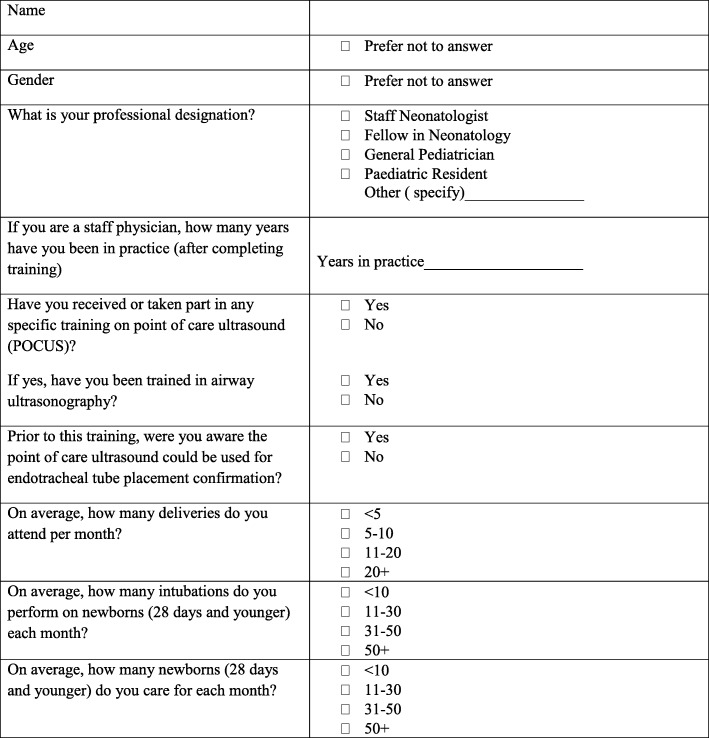
Learner Demographic Form

**Fig. 8:**
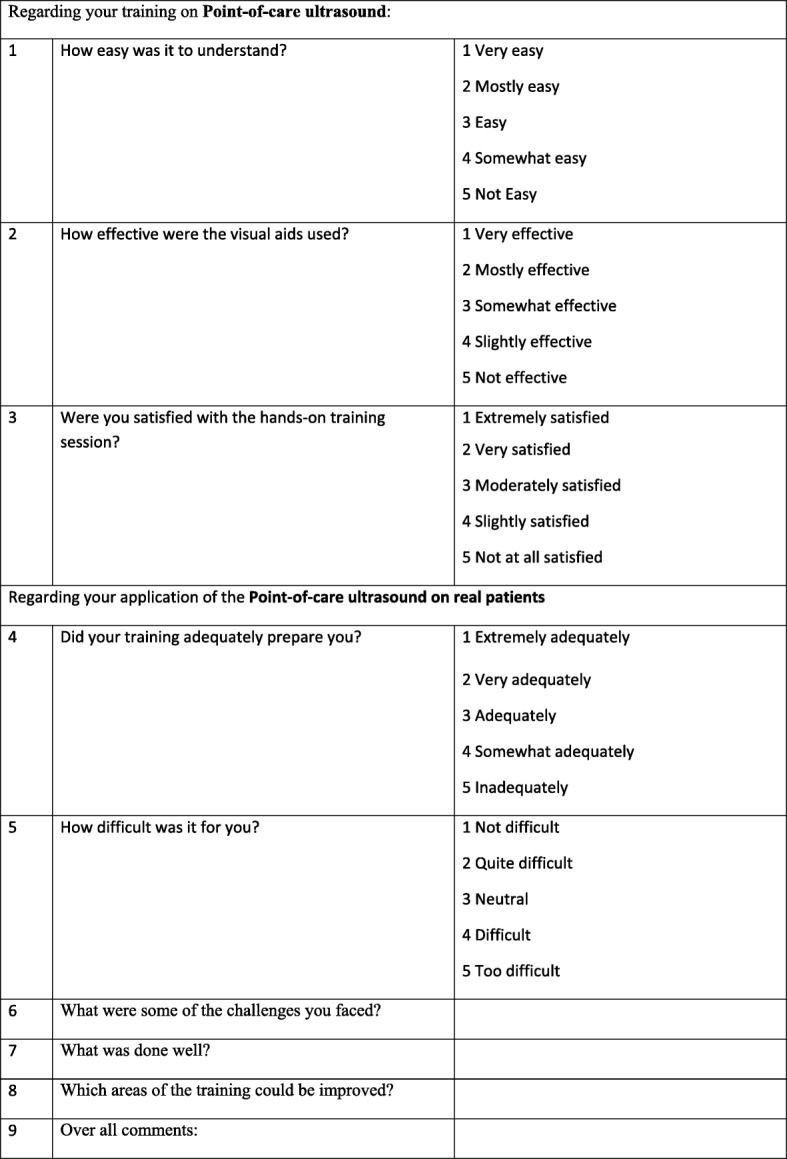
Learner feedback form

**Fig. 9:**
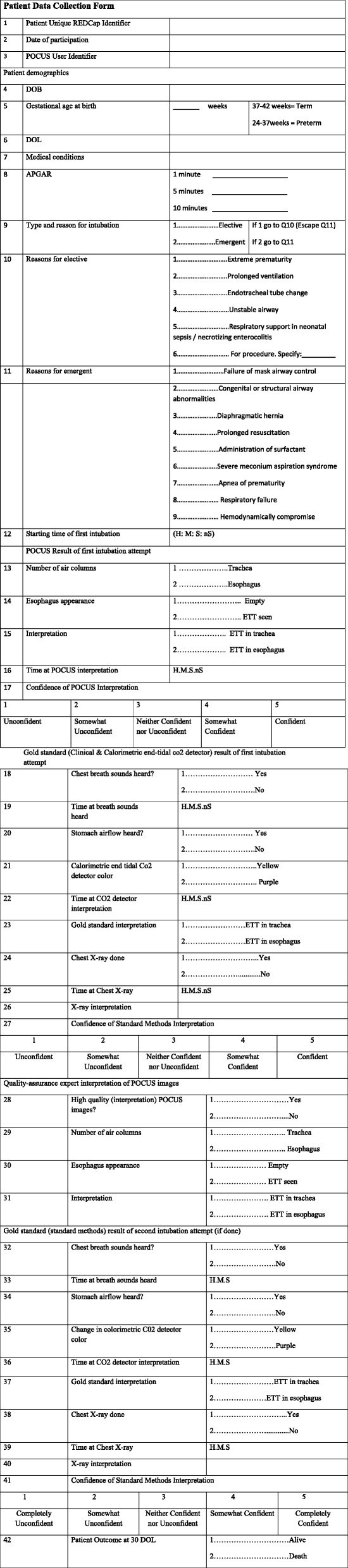
Patient Data Collection Form

Phase 1 data will be collected using the structured assessment of technical skills (see Fig. 5), the learner evaluation form (see Fig. 6), a learner demographic form (Fig. 7), and a learner feedback form (Fig. 8). These paper forms will then be transcribed into the Research Electronic Data Capture (REDCap) online tool. Phase 2 data will be collected by experienced female nurses who will be employed and trained for the study. Female nurse will be hired due to cultural acceptability, allowing access to the LR/OR. The data collector nurses will operate independently of both the clinical and POCUS teams, and will be available 24 h each day. They will initially collect intubation data using a patient data collection form (Fig. 9), then immediately transcribe the forms into REDCap.

For quality control, each POCUS examination will be recorded as a deidentified Mpeg4 video file on the POCUS tablet. These files will be sent via a secure online file transfer program to a non-AKUH paediatric intubation POCUS expert who was not part of study interventions and is blinded to any patient details. The expert will rate each video for high or low quality images, and will record an interpretation of esophageal or tracheal intubation.

### Measurement of outcomes

The primary outcome is diagnostic accuracy [(true positives+ true negatives)/total N] of POCUS (performed by healthcare providers with a brief education in intubation POCUS) for identifying tracheal versus esophageal intubation in newborns. The reference standard will be a composite of standard-of-care methods (auscultation, colorimetric capnography, and chest x-ray). The secondary outcome is the time difference between POCUS and standard-of-care methods in determining ETT location. The tertiary outcomes include the level of agreement between POCUS users and standard-of-care methods, the level of agreement between POCUS users and a POCUS expert, and the level of agreement between the POCUS expert and standard-of-care methods.

### Hypotheses

Neonatal health care providers trained on a novel ultrasound simulator in ETT localization will be > 95% accurate, compared to an external POCUS expert, when performing POCUS on intubated neonates, and will produce a result in less time than the current standard-of-care methods for ETT localization.

### Sample size

Phase 1 will involve 60 healthcare providers who attend deliveries and are exclusively involved in newborn care, which include 6 neonatal attending physicians, 8 neonatal fellows, 38 postgraduate medical trainees, and 8 senior nursing staff. These providers are all skilled in standard-of-care methods for ETT confirmation. Each 2-hour training session involves 15 participants, and the sessions are conducted 4 times.

The phase 2 sample size calculation indicates that a total of 292 neonatal intubations are required. There are approximately 5000 deliveries per year at AKUH, of which approximately 12% (600) of these newborns are admitted to the NICU. Approximately 70% (420) of these patients require intubation in a given year. The rates of incorrect esophageal intubation reported in the most recent multi-center study ranged from 1 to 12% [13]. Using a conservative rate of 5%, the sample size was calculated for the primary outcome (accuracy) using a two-sided equivalence test for correlated proportions. The standard proportion of correct tracheal intubation was assumed to be 95% and the maximum allowable difference between POCUS and standard-of-care methods was set to 0.05 (*α)*. To achieve 80% power at a 5% significance level, a total of 222 participants are needed for the primary outcome.

For the secondary outcome (time difference between POCUS and standard-of-care methods), using a conservative time difference of 5 s, we estimate a total sample size of 252 newborns will be needed to achieve a two-sided alpha of 0.05 and power of 80%.

Sample size estimates for the tertiary outcome are based on the kappa (κ) statistic for measuring agreement between POCUS and standard-of-care methods. Assuming 95% true agreement and a kappa statistic of 0.90, a sample size of 292 subjects results in a two-sided 95% confidence interval with a width of 0.100 (i.e. k = 0.85–0.95) using the Cohen’s large-sample formula for standard deviation of κ.

Therefore, to achieve all study objectives, a minimum sample size of *n* = 292 will be targeted.

### Timing

We estimate that both study phases can be completed in approximately 12 months.

### Data processing and analysis

Key study variables will be summarized univariately using means/standard deviations/histograms and frequencies/proportions as appropriate.

We will calculate sensitivity, specificity, positive and negative predictive values, positive and negative likelihood ratios, and the κ statistic (all with 95% confidence intervals) using standard formulas for a binomial proportion.

The mean time difference between POCUS and standard-of-care methods will be analyzed using paired sample t-tests. If the time distribution is skewed, we will examine median time differences using Wilcoxon signed-rank tests for matched data.

Data analysis will use SPSS statistical software, with the type 1 error rate set to 0.05. A subgroup analysis of premature newborns status will be performed as sample size permits. We will also analyze user accuracy as a function of number of POCUS exams performed during the study. An ad-hoc power analysis will be performed on stratified effects at study completion.

## Discussion

Intubation during newborn resuscitation is a critical life-saving skill that is difficult to learn and perform. Unsuccessful intubation can lead to devastating consequences, and one of the most common reasons for intubation failure of is esophageal placement of an ETT [26]. Ultrasound provides a simple, safe and effective method of differentiating between esophageal and tracheal intubations, but has not yet achieved widespread use in newborn resuscitation [27].

To our knowledge, only two previous studies have utilized ultrasound in real time for the confirmation of ETT position with comparison to standard-of-care methods [28, 29]. The first study included 16 NICU intubations and found a sensitivity of 92% and a specificity of 100% for ultrasound compared to capnography. Ultrasound was significantly slower than capnography, but significantly faster than x-ray [28]. The second study included 10 NICU intubations and 100% sensitivity and specificity for ultrasound compared to capnography, with ultrasound results generated significantly faster than x-ray [29]. Our study represents the largest study to date examining the use of POCUS for newborn intubations.

The second major strength of this study is the development and validation of a novel ultrasound simulator. Using materials costing only $2, an additional potential benefit of this study is to demonstrate that this simulator can be used to train healthcare providers in this important ultrasound skill. Simulators have the advantage of allowing providers to learn and practice skills prior to their application on patients, and other ultrasound simulators have been designed for this purpose [30]. Additional strengths of this study include 1) recorded images with quality assurance 2) different types of healthcare workers involved in being trained 3) extended follow-up period to measure skills retention, and 4) empowering health care providers to read and interpret images in real time thereby saving critical time and need for specialized human resources (radiologists).

The major limitation of this study is related to ultrasound cost as it pertains to the generalizability of the study. Although the simulator is low-cost, the ultrasound equipment is expensive and will not be able to be purchased in many low and middle-income settings at current price points. Over time, however, this will change as ultrasound technology continues to be more accessible. It is also important to note that ultrasound has a wide variety of applications in the NICU [31], and will increasingly become available for use.

## Data Availability

Given the sensitive nature of the data which includes ultrasound video clips of newborns, the data is only available to two co-authors (MT and KQA) via secured file sharing platform housed at The Hospital for Sick Children. The authors plan to publish aggregate study results in peer-reviewed journals and present the findings at conferences.

## Abbreviations

AKUH: Aga Khan University Hospital
CO2: Carbon dioxide
ETT: Endotracheal tube
LR/OR: Labour room/operating room
NICU: Neonatal intensive care unit
POCUS: Point of care ultrasound
SD: Standard deviation
